# Experimental insights into the azotosome hypothesis in Titan’s lake fluids

**DOI:** 10.1126/sciadv.aed1426

**Published:** 2026-03-11

**Authors:** Tuan H. Vu, Robert Hodyss

**Affiliations:** Jet Propulsion Laboratory, California Institute of Technology, Pasadena, CA, USA.

## Abstract

Since the discovery of hydrocarbon lakes and seas on Saturn’s moon Titan, there has been much speculation on whether these could serve as suitable environments to host exotic life. A decade ago, molecular dynamic simulations suggested that amphiphilic cyanide species such as acrylonitrile could self-assemble in these cold, nonpolar liquids to form stable closed membranes known as azotosomes, potentially compartmentalizing complex biochemical reactions. A subsequent thermodynamic study in 2020, however, concluded that azotosomes cannot exist under Titan’s conditions. Motivated by these incongruent computational results, this work undertakes the first experimental test of the azotosome hypothesis where we characterize acrylonitrile-methane and acrylonitrile-ethane mixtures under simulated Titan conditions using a combination of differential scanning calorimetry and Raman microscopy. The results indicate that acrylonitrile forms a stable molecular cocrystal with ethane while exhibiting little changes in the presence of liquid methane under experimental timescales. These findings suggest that the acrylonitrile-based azotosome structure would be unlikely to form in Titan’s lake fluids.

## Teaser

Acrylonitrile likely forms a cryomineral within Titan’s lake fluids rather than a vesicle structure akin to terrestrial liposome.

## INTRODUCTION

Saturn’s largest moon Titan is one of the most earthlike bodies in the solar system, with a dense (~1.5 bar) atmosphere composed mostly of nitrogen (~95%), as well as standing lakes and seas on its surface. These surface liquids are made up primarily of methane and ethane, forming a meteorological cycle analogous to Earth’s water cycle (*1*). Titan also features winds and weather systems of rain, clouds, and seasonal changes (*2*–*4*), shaping a landscape that resembles Earth in form. These similarities to our planet have spurred scientific interest in this icy world for decades, including its potential to host exotic life despite the extreme surface temperatures (~90 to 94 K). The NASA Dragonfly mission, currently due to launch in July 2028, will carry a rotorcraft lander to perform measurements at various sites on Titan’s surface. While not specifically designed to be a life detection mission, Dragonfly will investigate Titan’s intriguingly complex organic materials and provide a better understanding of the chemical processes that could lead to habitable environments (*5*).

The presence of solvents such as liquid methane and ethane provides an enticing medium that can support chemical reactions, albeit at low temperatures. In 2015, a seminal molecular dynamic (MD) simulations study (*6*) suggested that certain types of cyanide species that are present in Titan’s atmosphere, particularly acrylonitrile [which has been confirmed spectroscopically by Atacama Large Millimeter/submillimeter Array observation (*7*)], could self-assemble in such cold, nonpolar liquid environments to form stable, spherical membranes known as azotosomes. These structures, composed of acrylonitrile’s polar head groups on the inside and vinyl tails facing the solvent on the outside, were proposed as an alternative analog to lipid bilayer membranes, whose formation is a key step in the evolution of terrestrial life (*8*–*10*). The MD results predicted that azotosomes have good stability, high energy barrier to decomposition, and an elasticity in cryogenic solvents that is comparable to that of lipid bilayers in water at room temperature. Consequently, these structures could encapsulate chemicals long enough to allow for interactions, thus presenting a potential for exotic biochemical reactions to be compartmentalized.

A subsequent quantum mechanical calculation study (*11*), however, cast doubt on this intriguing idea. Thermodynamic stability estimates under the conditions of Titan indicated that supramolecular aggregates of acrylonitrile would favor its crystalline solid form over the azotosome structure by ~8 to 17 kJ/mol (for reference, thermal energy at 90 K is 0.75 kJ/mol). The prospect for azotosome formation is further complicated by the fact that thousands of acrylonitrile monomers would need to come together for its formation, while the solubility of small polar molecules are expected to be very low in cryogenic liquid methane (*12*). Accordingly, these authors concluded that azotosomes would be unstable and unlikely to form under Titan’s conditions.

These opposing modeling outcomes naturally prompt the need for laboratory investigation, yet such work has not been conducted to date. We present here the first attempt to experimentally assess the viability of the azotosome hypothesis by characterizing the chemical behavior of acrylonitrile in liquid methane and liquid ethane under Titan-relevant conditions. Specifically, we simulate the scenario in which acrylonitrile, produced in the upper atmosphere via photochemistry (*13*, *14*), precipitates as a solid that subsequently descends onto the surface and comes into contact with the lake fluids. Our experimental approach uses a combination of two analytical methods: differential scanning calorimetry (DSC) and Raman microscopy. DSC is a commonly used tool in the pharmaceutical industry for characterizing vesicle formation (*15*–*17*), while Raman offers a valuable probe of the changes in the molecular environment of compounds at high spatial resolution in a nondestructive manner. Together, these two complementary techniques will help elucidate the changes in the phase behavior of acrylonitrile and any resulting product from interaction with the cryogenic hydrocarbon solvents. Understanding this chemistry is crucial for evaluating the likelihood of azotosomes and consequently providing important insights into this aspect of Titan’s astrobiological potential.

## RESULTS

### Acrylonitrile-ethane system

**Fig. 1.**
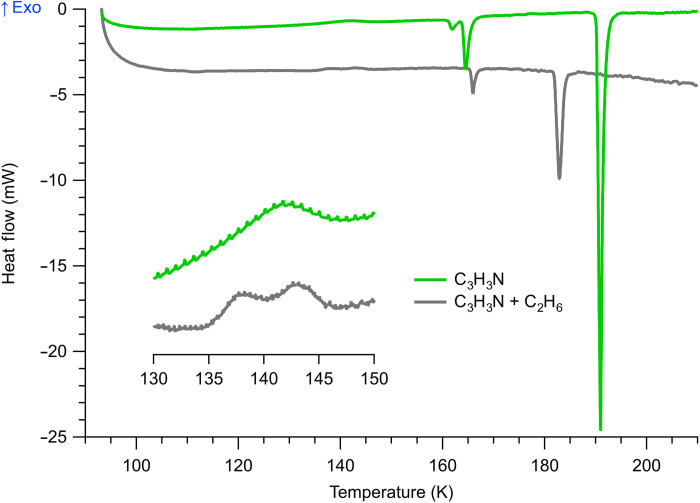
DSC thermograms of acrylonitrile and acrylonitrile + ethane (10 wt % mixture) upon heating from 94 K at a rate of 0.15 K/min. Positive heat flows (upward peaks) indicate exothermic transitions. The doublet at 138 and 143 K (inset) together with depressed bulk melting in the mixture is suggestive of a cocrystalline phase between acrylonitrile and ethane. exo, exothermic.

Because of easier experimental handling of liquid ethane (e.g., lower volatility and larger liquid range) compared to liquid methane, the acrylonitrile-ethane system was investigated first. Figure 1 shows the DSC thermogram of this system (gray trace) obtained upon heating from 94 K at a rate of 0.15 K/min, in juxtaposition with that of pure acrylonitrile (green). Looking at the latter, it is evident that acrylonitrile itself exhibits certain degrees of polymorphism. Three distinct crystalline phases were found to be present, as indicated by their sharp endothermic (melting) transitions respectively at 161.2, 163.9, and 190.2 K, the latter of which is the most intense and corresponds to the known bulk melting point of acrylonitrile. On closer inspection, these transitions were preceded by a small exothermic peak at 142 K (inset), likely due to crystallization of an initially amorphous or disordered phase. The enthalpy of this peak is −2.71 J/g, which appears to correlate to the solid-state transition to the metastable phase that melted at 161.2 K (Δ*H* = 2.88 J/g). This polymorphism is a common behavior among many small organic compounds (*18*–*20*).

In the presence of ethane, acrylonitrile shows a markedly different thermogram (Fig. 1, gray trace). Here, the three crystalline phases previously observed in solid acrylonitrile have collapsed into two phases, with onset temperatures of 165.5 and 181.9 K (Table 1). The latter feature, in particular, represents a substantial −8.3-K shift compared to the bulk melting point of pure acrylonitrile. This peak is not due to evaporation of liquid ethane (nominal boiling point of 185 K at 1 bar), as this process was not observed in our experiment due to the enclosed nature of the DSC cell. This melting point depression is therefore suggestive of “impurities” in the acrylonitrile, most likely by incorporation of ethane into its crystal lattice. These melting point shifts in the acrylonitrile-ethane system are accompanied by an exothermic doublet at 138 and 143 K (inset), a behavior not seen in the acrylonitrile control. These evidence together point to the occurrence of a chemical transformation, potentially resulting in the formation of a binary crystalline structure between the species.

**Table 1.** Transition temperatures of acrylonitrile as observed in the pure solid and in mixtures with liquid methane and liquid ethane. Both onset (start of thermal event) and peak temperatures (maximum heat flow) are reported for sharp transitions typically exhibited by crystalline materials. For thermal events that yield asymmetric peaks (where onsets are not well defined), only the peak temperatures are reported.

System	Type of transition	(Onset)/peak temperatures (K)
C_3_H_3_N	Exotherm	142
	Endotherm	(161.2)/161.9
		(163.9)/164.5
		(190.2)/191.0
C_3_H_3_N + C_2_H_6_	Exotherm	138
		143
	Endotherm	(165.5)/166.0
		(181.9)/182.9
C_3_H_3_N + CH_4_	Endotherm	(161.0)/161.6
		(164.4)/164.9
		(188.5)/189.7

**Fig. 2.**
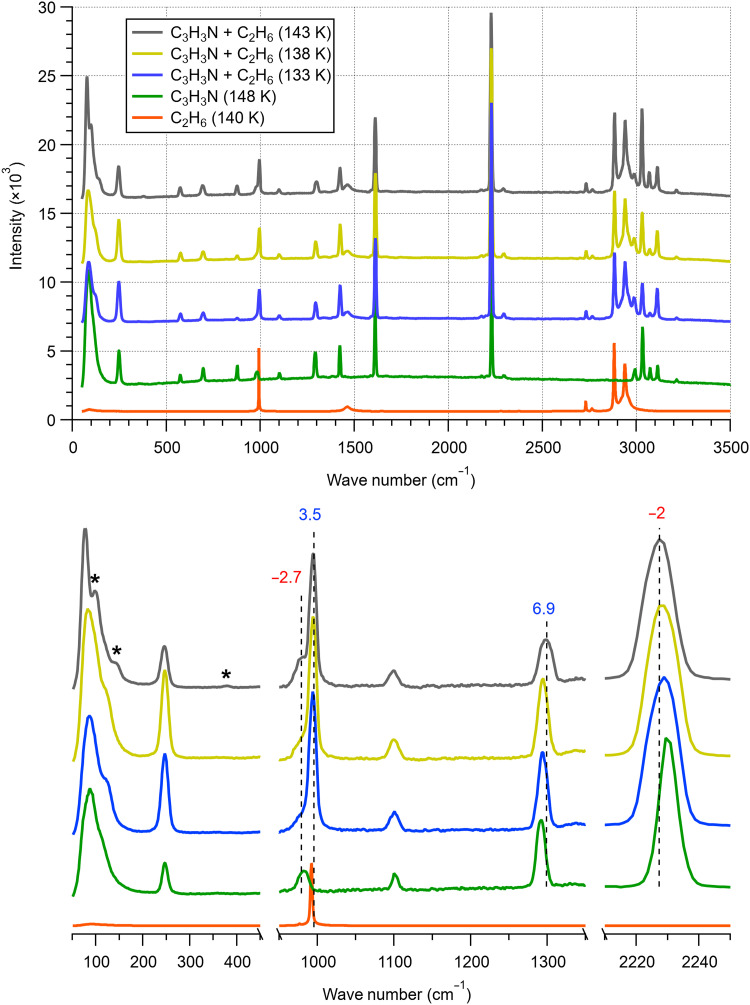
Raman spectra of the 10 wt % acrylonitrile-ethane mixture in comparison with those of the constituents. (**Top**) Full range and (**bottom**) zooming in different regions of interest. The dashed lines denote the major peaks in the spectrum of the mixture at 143 K (gray), with associated numbers indicating the magnitude of spectral shift compared to the pure components (positive values denote blue shifts and negative values denote red shifts). Stars mark peaks in the lattice region that are not seen in the controls.

To elucidate the chemical environments within the acrylonitrile-ethane mixture, we performed Raman experiments, focusing on the temperature range where the phase transitions were observed in the DSC measurement. Figure 2 displays the spectra obtained during the crystallization exotherms at 138 and 143 K, as well as one before these events at 133 K for context (blue trace). Several spectral changes are evident, especially at 143 K (gray trace). In particular, the low frequency region (representing the lattice vibrations) becomes distinctly more resolved, with two prominent features around 97 and 143 cm^−1^. Acrylonitrile itself (green trace) does not exhibit such a structure, and ethane (being a liquid) has essentially no contribution in this spectral region. This observed transformation in the lattice modes is thus consistent with an additional ordered solid phase being formed in the sample. There is also a weaker but visible peak at 379 cm^−1^ that is not present in either of the pure components, supporting the interpretation of a mixed phase.

In addition to the emergence of these features, acrylonitrile and ethane exhibit notable spectral shifts in several of their vibrational signatures, both in the red and blue directions (Fig. 2, bottom). Specifically, the C ≡ N stretch at 2227 cm^−1^ and C-H wagging modes at 980 cm^−1^ (*21*) undergo a ~2- to 3-cm^−1^ red shift compared to pure solid acrylonitrile, while the C-H rocking mode at 1299 cm^−1^ experiences a blue shift of ~7 cm^−1^ in the presence of ethane. For ethane, the C-C stretch is observed at 995 cm^−1^ in the mixture, a sizeable 3.5-cm^−1^ blue shift from that of the liquid (shifts of the similar magnitude are also seen for the C-H stretching modes around 2800 to 3000 cm^−1^). These changes, together with the DSC results, signify a different molecular environment for both species. On the basis of similar behavior that has been observed in many other organic systems (*22*–*24*), we ascribe this to the formation of a cocrystalline structure whereby ethane is incorporated or confined within the acrylonitrile lattice.

**Fig. 3.**
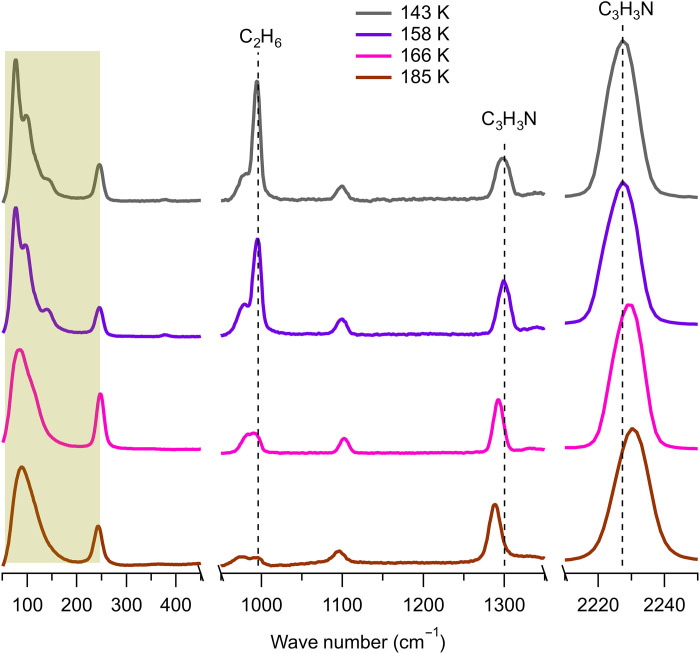
Thermal stability of the acrylonitrile:ethane cocrystal. Yellow box highlights the lattice vibration, which shows an obvious change upon cocrystal dissociation at 166 K. This is accompanied by spectral shifts of both acrylonitrile and ethane signatures (dash lines) reverting back to their respective solid and liquid states.

We subsequently examine the thermal stability of the presumed acrylonitrile:ethane cocrystal. The DSC thermogram shows no transition between 143 and 166 K, where an endothermic event took place. This is corroborated by the corresponding Raman spectra in Fig. 3. Following cocrystal formation at 143 K (gray trace), the spectrum remains stable throughout this temperature regime, as shown with the intermediate spectrum at 158 K (purple trace). At 166 K, however, the cocrystal exhibits several spectral changes, most noticeably in the lattice region. The ordered structure previously seen for the cocrystal (highlighted in yellow) and the 379 cm^−1^ peak have disappeared entirely. The C-C stretch of ethane at 995 cm^−1^ and the C-H bending and C ≡ N stretching modes of acrylonitrile (dashed lines) have also reverted to their frequencies in liquid ethane and solid acrylonitrile phases, respectively. These changes confirm that the transition at 166 K in the DSC thermogram is due to dissociation of the cocrystal. As the temperature is raised to 185 K, the remaining acrylonitrile melted and mixed with the dissolved ethane, leading to the depressed melting point compared to pure solid acrylonitrile. Overall, we did not observe behavior that would be consistent with the formation of azotosome [e.g., characteristically broad and asymmetric transitions in the DSC profile (*17*)]. Instead, the experimental evidence points to a cocrystal (stable up to 166 K) between the two species.

### Acrylonitrile-methane system

**Fig. 4.**
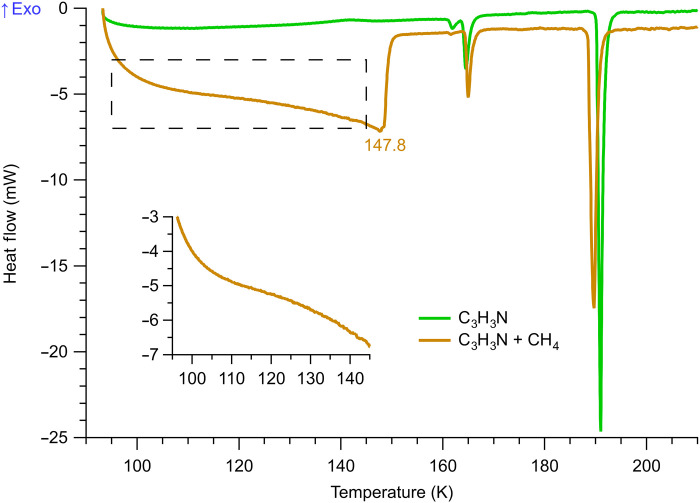
DSC profiles of acrylonitrile and acrylonitrile + methane (10 wt % mixture) upon heating. The broad endotherm between 95 and 150 K is due to gradual evaporation of liquid methane. Inset shows an expanded view of the boxed region.

We next examined the acrylonitrile-methane mixture. Its DSC thermogram, displayed in Fig. 4, exhibits a long endothermic tail stretching from 95 to 150 K. In contrast with sharp melting transitions of pure crystalline materials, this type of profile typically signifies a gradual process that absorbs heat over a wide temperature range, such as evaporation of a solvent. In this case, that process would correlate with the evaporation of liquid methane, as the sample was slowly heated, resulting in an increase in pressure (the DSC cell is a sealed system) until all of the liquid was driven away. The peak temperature here was observed at 147.8 K, which corresponds to a methane vapor pressure of ~9.4 bar (*25*). After this point, the remaining acrylonitrile was seen to undergo a typical melting process as seen in the pure solid (green trace). All three previously observed acrylonitrile phases were present, albeit slightly shifted in temperatures (by ~1 K or less—Table 1) due to the self-generated pressure. The small magnitude of these shifts, together with the lack of any distinct endothermic or exothermic peaks for acrylonitrile, suggests that methane has little effect on its crystalline phases.

We further zoomed into the region between 95 and 145 K (inset of Fig. 4) to examine whether there were detectable thermal transitions that could have been due to the azotosome (or any other structure) before the evaporation of liquid methane. The heat flow in this range was also very smooth and featureless, without any visible peak. Thus, unlike its ethane counterpart, the acrylonitrile-methane mixture did not show any indication of a reaction or formation of a molecular complex at cryogenic temperatures, at least under our experimental timescales and conditions.

## DISCUSSION

The chemical behavior of acrylonitrile in the presence of liquid methane and liquid ethane under simulated Titan’s surface conditions was investigated in this work. DSC and Raman measurements both show strong evidence for the emergence of an acrylonitrile:ethane cocrystal that is stable to a relatively high temperature (166 K). The acrylonitrile-methane mixture, on the other hand, exhibits little specific interaction between the components. The former system is reminiscent of a recently reported cocrystalline phase (*24*) seen upon mixing of ethane and HCN, another polar nitrile species of great abundance on Titan (*13*, *26*). This lends further credence to our current interpretation, considering the chemical similarity between the two nitrile species. These two potential cocrystals, in conjunction with the well-studied benzene-ethane cocrystal (*23*, *27*–*29*) and ethane clathrate hydrates (*30*), now constitute four ethane-bearing solid materials that could exist under Titan’s conditions, demonstrating the versatility of this lake component to interact with a range of polar as well as nonpolar compounds. Ethane, hence, is gradually establishing itself to be more than just a solvent but an active participant in chemical processes even at cryogenic temperatures. We did not observe direct evidence of the formation of acrylonitrile-based vesicles in this fluid (nor in liquid methane), at least under experimental timescales. Our null outcome corroborates previous quantum mechanical calculations (*11*) that predicated the inherent thermodynamic instability of the azotosome structure.

We note that our experimental settings only simulated the conditions in which solid acrylonitrile comes into contact with liquid methane and liquid ethane. Recently, a mechanism for vesicle formation via aerosol droplets on Titan has been proposed (*31*). In this scenario, amphiphilic cyanide molecules (such as acrylonitrile) formed in the atmosphere would be carried into the lakes via condensation or rainfall, resulting in a monolayer on the lake surface that is held together by dipole interaction. Methane rain droplets then cause local splashing, generating a mist of secondary droplets that retain the surface monolayer. As these coated secondary droplets resettle onto the lake surface, their monolayer would combine with the bulk monolayer into bilayers and form temporarily stable vesicles that could gain thermodynamic stability as they grow over time. While this postulated pathway remains to be verified experimentally, it is worth considering a few possible complicating factors. Namely, Titan’s lakes and seas are composed primarily of mixtures of methane and ethane (plus propane and other minor solutes) (*32*). Although the relative proportions of these components can vary depending on location, seasonal changes, or stratification dynamics (*33*, *34*), which may lead to enriched methane content, they are unlikely to ever be free of ethane. Hence, there would always exist the potential for an acrylonitrile “sink” via the cocrystal formation process, as shown in the present work. In addition, while some Titan’s seas (such as Ligeia Mare) might have acrylonitrile abundance that reaches saturation (*7*), the estimated mole fraction is rather low (2.2 × 10^−5^) (*35*), placing a stringent limit on the initial amount that could be dissolved in solution. These factors together would considerably hinder the ability of acrylonitrile molecules to accumulate on the lake surface and the subsequent steps in the aerosol droplet mechanism.

Although detailed formation kinetics of the acrylonitrile:ethane cocrystal is not examined in this work, previous experiments on a similar system (benzene:ethane) have indicated that the process is rather rapid (less than a day to reach completion at 90 K) (*28*). Thus, the uptake of acrylonitrile by ethane within the lakes would likely be favorable both thermodynamically and kinetically. Besides cocrystallization, the pure solid phase of acrylonitrile is another stable state under the environmental conditions of Titan (*11*). Therefore, if the azotosome structure were to exist, then it would need to contend with (and thermodynamically overcome) both the cocrystal and the solid phases. The feasibility of the aerosol droplet pathway for vesicle formation may eventually come down to the kinetics of these various competing processes at cryogenic temperatures.

In summary, our work shows that acrylonitrile, a candidate for self-assembled vesicles in Titan’s lakes and seas, could be readily removed from solution by the ethane present in the fluids via cocrystal formation. This finding further reduces the prospect for vesicles on Titan from a thermodynamic standpoint, but it does not rule out the possibility that they might exist temporarily or be transiently stable. Further experimental investigations are thus warranted, especially in addressing the kinetic aspect and the aerosol droplet mechanism using techniques such as dynamic light scattering combined with surface-enhanced Raman spectroscopy (*31*). Regardless of whether Titan has ever harbored vesicles, the chemistry of these systems has deepened our understanding of the history and distribution of organic compounds on this body. One often encountered argument against Titan’s surface as a habitable environment is the presumed slow rate of chemical reactions at cryogenic temperatures. However, current and many previous works (*23*, *24*, *27*–*30*, *36*–*42*) have showcased intriguing, unexpected chemistry that is not only possible but also enabled by such frigid environments. Titan’s surface habitability thus remains an intriguing notion, whether by way of vesicles or other means.

## MATERIALS AND METHODS

For the DSC measurements, 10 wt % mixtures of acrylonitrile (Sigma-Aldrich) in liquid methane and liquid ethane (Matheson Tri-Gas, ultrahigh purity 99.95%) were prepared by depositing the appropriate amounts into a stainless steel sample cell (8.5-ml capacity) immersed in liquid nitrogen. The total volumes of the samples were 2 and 1.5 ml for the acrylonitrile-methane and acrylonitrile-ethane mixtures, respectively, owing to the difference in density between liquid methane and liquid ethane. The sample cell was sealed by compressing a nickel gasket at 35-N·m torque and subsequently transferred to a Setaram BT 2.15 Calvet-type differential scanning calorimeter, which contained an identical cell that was kept empty for reference. Each cell is connected to a thermopile equipped with a three-dimensional array of 64 thermocouples to measure the temperature differential between them over time, from which the heat flow can be obtained. The temperature profile of the experiment was preprogrammed using the accompanying Calisto software, which entailed an initial cooling step to 94 K and held over a 24-hour period, followed by a heating ramp at 0.15 K/min to 295 K. A slow heating ramp is used to minimize errors introduced by heat transfer hysteresis between the furnace and the calorimetric cells (*43*).

For the Raman measurements, a 10-μl aliquot of acrylonitrile was deposited onto a well of a 5-mm-thick microscope slide positioned inside a cryogenic optical stage (LTS350, Linkam Scientific Instruments Ltd.) held at 273 K to minimize evaporation. Experiments were conducted under one atmosphere of N_2_ gas, simulating Titan’s surface conditions. The cryostage’s temperature was then lowered to 93 K (average Titan’s surface temperature), and liquid ethane was deposited on top of the frozen acrylonitrile droplet. Subsequently, the cryostage was mounted onto an XYZ motorized translation stage (Märzhäuser Wetzlar GmbH & Co. KG) underneath the Olympus BXFM objective turret of a confocal dispersive Raman microscope (Horiba Jobin-Yvon LabRAM HR), and the sample was monitored through the cryostage’s viewing port. A frequency-doubled Nd:YAG laser emitting at 532 nm (Oxxius; 100-mW output power) was used as the excitation source for the Raman effect, with spectra collected via an LWD 50× lens at 1.5 cm^−1^ per pixel resolution using a grating of 600 grooves/mm. Frequency calibration was performed using the sharp peak at 520.7 cm^−1^ of a silicon wafer. At each temperature point, the sample was equilibrated for 5 min before Raman spectra were obtained. Spectra were collected with acquisition time of 2 s and two accumulations.
